# Quantitative neuropathological assessment to investigate cerebral multi-morbidity

**DOI:** 10.1186/s13195-014-0085-y

**Published:** 2014-11-28

**Authors:** Johannes Attems, Janna H Neltner, Peter T Nelson

**Affiliations:** Institute of Neuroscience, Newcastle University, Campus for Ageing and Vitality, Newcastle upon Tyne, NE4 5PL UK; Department of Pathology, Division of Neuropathology, University of Kentucky, 800 Limestone Street, Lexington, KY 40536-0230 USA

## Abstract

The aging brain is characterized by the simultaneous presence of multiple pathologies, and the prevalence of cerebral multi-morbidity increases with age. To understand the impact of each subtype of pathology and the combined effects of cerebral multi-morbidity on clinical signs and symptoms, large clinico-pathological correlative studies have been performed. However, such studies are often based on semi-quantitative assessment of neuropathological hallmark lesions. Here, we discuss some of the new methods for high-throughput quantitative neuropathological assessment. These methods combine increased quantitative rigor with the added technical capacity of computers and networked analyses. There are abundant new opportunities - with specific techniques that include slide scanners, automated microscopes, and tissue microarrays - and also potential pitfalls. We conclude that quantitative and digital neuropathologic approaches will be key resources to further elucidate cerebral multi-morbidity in the aged brain and also hold the potential for changing routine neuropathologic diagnoses.

## Cerebral multi-morbidity

It is becoming increasingly clear that, as a rule, the aging brain is characterized by the simultaneous presence of multiple neuropathological lesions rather than the hallmark lesion(s) of a single age-associated neurodegenerative disease [1]. Moreover, the prevalence of this cerebral multi-morbidity increases with age, and post-mortem studies indicate that, in brains of demented individuals over 80 years of age, the presence of only one, single disease is a rare finding [2-7]. More details regarding the prevalence of mixed pathologies can be found in the article by Rahimi and Kovacs in the present review series of *Alzheimer’s Research* & *Therapy* [8]. Alzheimer’s disease (AD) in particular often presents with comorbid processes, including cerebrovascular disease, Lewy body (LB) pathology, argyrophilic grain disease, transactivation response DNA binding protein 43 kDa (TDP-43) pathology, and hippocampal sclerosis, and about two-thirds of aged human brains contain substantial non-AD pathology [9-11]. Indeed, in AD that is neuropathologically characterized by amyloid-beta (Aβ) and tau pathology (hyperphosphorylated tau), LB pathology (α-synuclein) is present in up to 43% [1,12] (AD with LBs restricted to the amygdala is considered a distinct form of α-synucleinopathy [12]) and severe cerebrovascular lesions are observed in up to 20% [2] of cases, respectively. TDP-43 pathology often but not invariably restricted to the amygdala and granule cell layer of the dentate gyrus and entorhinal cortex is present in up to 57% [11,13-15], and recently Josephs and colleagues [15] demonstrated that TDP-43 is an important factor in the manifestation of clinico-imaging features of AD. In LB disease that is characterized by α-synuclein pathology, we found Aβ pathology in 95% of cases, considerable tau pathology (Braak stages V/VI) in 55%, and various degrees of cerebrovascular pathology in 75% [16]. Both Aβ pathology (semi-quantitative scores [17]) and tau pathology (Braak stages [18]) correlated with LB pathology, and co-localization between hyperphosphorylated tau and α-synuclein has been reported [12,17]. Pure vascular dementia without additional lesions is rare (for example, 12.3% in [4]), and frequently additional AD pathology is present. Whereas the common presence of neuropathologic comorbidities has been described in many autopsy series, the clinical diagnosis of multiple neurodegenerative pathologies in one single patient remains challenging and additional pathologies are often clinically unnoticed [19]. This may partially be due to a lack of clinico-pathological correlative studies that identified subtle clinical signs and symptoms that could point toward additional concomitant pathologies.

## Quantitative neuropathological assessment

Clinico-pathological correlative studies are frequently based on semi-quantitative data and ordinal-type parameters to define the amount of pathology present in a given post-mortem brain. These semi-quantitative data are usually provided on standardized four-tiered ordinal scales: absent, mild, moderate, and severe (for example, for tau [20] and α-synuclein [21]). Although such semi-quantitative data are very useful for providing the neuropathological diagnosis, they often inaccurately reflect the actual amount of pathology present and this has major implications when data from large clinico-pathological correlative studies are entered into databases, since cases that might actually differ quite considerably regarding the amount of pathology fall into the same category. For example, we found that the amount of tau pathology in cases semi-quantitatively scored ‘severe’ differed significantly when the actual area covered by immunopositivity was measured [1]. It is likely that new clinico-pathological phenotypes more accurately reflecting cerebral multi-morbidity would be identified by assessing the amount of pathology in a more quantitative way.

Indeed, in quantitatively assessing entorhinal and hippocampal tau pathology in a large cohort (n = 889) of both clinically and neuropathologically diagnosed AD cases, Murray and colleagues [22] identified typical AD as well as hippocampal sparing and limbic predominant subtypes of AD. When comparing their quantitative neuropathological data with clinical findings, the authors found that these subtypes of AD differed in clinical presentation, age at onset, disease duration, and rate of cognitive decline from typical AD [22]. In a subsequent study, the authors found that magnetic resonance imaging (MRI) could predict these subtypes during life since hippocampal sparing AD showed the most severe cortical atrophy while the most severe medial temporal atrophy was observed in limbic predominant AD [23]. Of note, these AD subtypes and their associations with cortical atrophy in MRI would not have been identified if only semi-quantitative methodologies were employed, since all cases showed ‘severe’ entorhinal tau pathology. Only by the use of quantitative measurements did the ‘severe’ group show differences in the actual amount of pathology present. The authors more recently demonstrated that limbic predominant AD differed from neurofibrillary tangle (NFT) dominant dementia as the latter showed significantly less tau pathology in the mid-frontal cortex [24]. The authors also suggested that, in hippocampal sparing AD, tau pathology may begin in the neocortex, since they found a fourfold increase in the amount of neocortical late-stage tau (antibody Ab39 to a conformational epitope in NFTs detecting late-stage tangles [25]) in hippocampal sparing AD compared with typical AD [24]. The findings from two large clinico-pathological correlative studies - the Nun Study and the Adult Changes in Thought (ACT) Study - indicated that 12% (Nun Study) and 8% (ACT Study) of non-demented subjects showed severe AD pathology reflected by Braak stage V-VI. However, quantitative assessment of NFT in both frontal and temporal cortices revealed that these non-demented subjects showed less NFT compared with demented subjects with Braak stage V-VI [26] and demonstrated a considerable range of pathology within Braak stage VI [27]. It is important to note that true ‘end-stage’ neurofibrillary pathology, measured with quantitative methods, has never been associated with an individual patient with ante-mortem intact cognition [27]. The density of neuritic plaques and NFTs rose significantly as a function of severity of dementia in subjects who were 60 to 80 years old, but no such association was found when subjects were over 90 years old [28], suggesting that additional factors contribute to the development of dementia in the oldest old.

The examples given above clearly indicate that quantitative neuropathological assessment allows the identification of clinico-pathological associations that are not detected by using semi-quantitative assessment alone. Moreover, given that quantitative assessment of tau pathology in AD cases points toward new clinico-pathological phenotypes [22], we assume that quantitative assessment of various neuropathological lesions in large autopsy cohorts would be beneficial to further elucidate possible mutual relationships between pathologies as well as their combined influence on the clinical picture. Hence, large clinico-pathological correlative studies could identify subtle clinical features that point toward underlying pathologies. However, manual methods of quantitative assessment are time-consuming since they involve either manual inspection of histological slides with visual counting of pathological lesions or importing individual images into an image analysis system for further analysis. Hence, automated methods for quantitative assessment might be helpful to investigate large study cohorts and to perform quantification in a routine setting.

## Automated quantitative neuropathological assessment

Of note, the aim of this section is not to provide a detailed methodological description (which is outside the purview of this review article) or a comprehensive summary of all systems that might be currently used in other centers. Rather, we aim to give an overview of two methodologies for automated quantitative assessment that are currently used in our own laboratories, and we refer to our own published studies that successfully employed these methods.

### Slide scanner and digital pathologic image analysis

**Figure 1:**
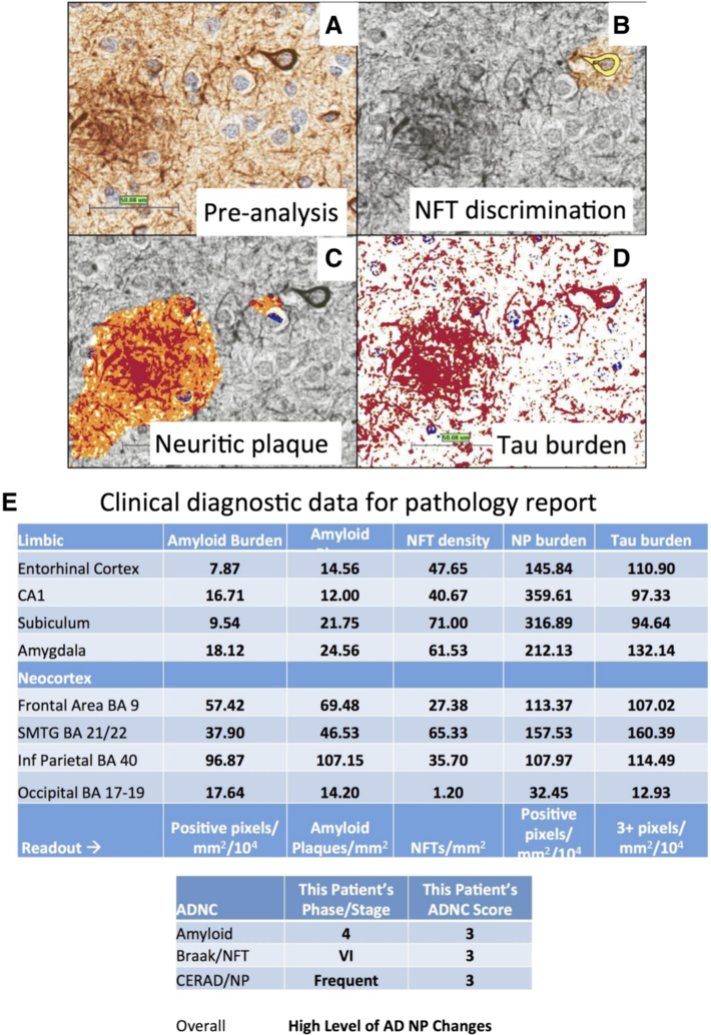
**Digital quantification of tau pathologies. (A)** Analyses are performed on a paired helical filament-1 immunostained section. **(B)** After the crafted Genie neurofibrillary tangle/neuritic plaque (NFT/NP) algorithm was used to isolate the NFTs, the NFT density (NFTs/mm^2^) is determined by a modified nuclear algorithm, with NFT pseudo-colored yellow. **(C)** In a similar manner, the NP burden is calculated by first using the same crafted Genie algorithm to isolate the NPs, here pseudocolored orange. **(D)** An overall tau burden is also calculated: red: positive immunohistochemical (IHC) staining; blue: negative IHC staining. Scale bar, 25 μm. **(E)** A sample of the data that are provided in each pathology report from the University of Kentucky for a quantitative description of Alzheimer’s disease (AD) pathology. ADNC, Alzheimer’s disease neuropathologic change; CA1, hippocampus sector CA1; CERAD, Consortium to Establish a Registry for Alzheimer’s Disease; Inf, inferior; SMTG, superior and middle temporal gyri.

**Figure 2:**
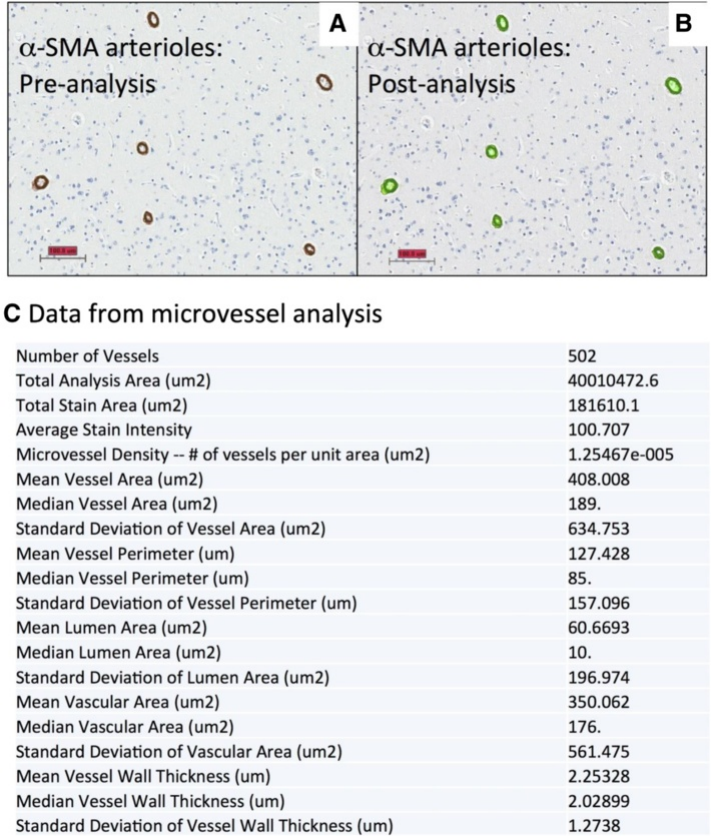
**Digital quantification in a brain section using alpha-smooth muscle actin (α** **-SMA) immunohistochemistry which labels arterioles.** Actual immunohistochemical stain is brown **(A)** whereas the digital detection of those markers is pseudocolored green **(B)** after digital analyses were run. This is the basis for further *in silico* analyses of the blood vessel morphology. Scale bars, 100 μm. **(C)** A sample of the data that are collected from each analysis. Note that aspects of the blood vessel lumen, in addition to the lumen wall, are measured in a systematic way.

Digital pathology offers a valuable resource for quantitative pathology in neurodegenerative disease. In the recent consensus recommendation article sponsored by the National Institutes of Health and the Alzheimer’s Association [29], it was noted that ‘both quantitative and qualitative aspects of AD neuropathologic change have significance, but current diagnostic methods are not robustly quantitative and/or not systematically qualitative’. This statement confirms that more quantitative diagnostic methods are required in both the clinical and research settings. Toward those goals, digital pathology offers multiple benefits that surpass both semi-quantitative methods and manual counts. Digital algorithms offer a superior reproducibility and higher throughput performance that could enable a far more standardized approach to the assessment of AD neuropathologic changes (ADNCs). If individual centers began to use a standard algorithm for quantification, results could be used across institutions, exponentially increasing the statistical power available to all centers involved. Among the options for returning quantitative changes in pathologies, the digital approach is relatively efficient when it comes to manpower. With these algorithms, more pathology can be counted faster and more reproducibly than by manual inspection alone. In addition, more parameters can be rigorously examined, from staining intensity to plaque size and more as described below. As our use of this technology advances, it will open up a new understanding of the pathologies in human brain aging. Here, we provide some examples of results derived from the Aperio ScanScope (Leica Biosystems, Nussloch, Germany), which is used routinely at the University of Kentucky to document pathologic changes, including both neurodegenerative disease pathology and other subtypes of brain disease, that affect older persons. Over 100 slides can be scanned automatically in a batch. Although it does take additional time to scan the slide and set up the analysis windows (approximately 45 minutes to prepare and scan at 40× via the semi-automated method and an additional 510 minutes to select the analysis windows per slide), the bulk of the analysis work is done by the server alone. These analyses can be set up during the day and then allowed to run overnight without interruption (120 slides per night). In addition, neuropathologic expertise is not a requirement for this method, and we found that workers at various phases of training could all come up with very similar results in scoring ADNC quantification [30]. As the analysis algorithms are held constant, regardless of who sets up the windows, the data are consistent. In future endeavors, this could be expanded to involve algorithm sharing between institutions and thus improve the inter-rater reliability between the different research centers to help standardize the field of quantitative ADNCs. In specific applications, digital pathology can be used for both novel discoveries and routine clinical duties. For example, although manually quantified ADNC numbers suggested that Aβ plaque burden leveled off with increasing neurofibrillary pathology [31,32], we failed to identify that it actually decreases with increasing tau burden by our manual methods alone. However, this phenomenon could be well demonstrated with digital methods [30]. Additionally, it has previously been shown that apolipoprotein ε4 alleles correlate with increased Aβ plaque pathology [33-36]; however, we could demonstrate with digital methods that the plaque burden was partly related to larger plaques, not just more plaques [30]. These data may enable other new insights into the pathologic changes seen in AD. In addition to the benefits for research, these data may be appreciated by clinicians who may desire more than a semi-quantitative idea about neuropathologic burden. Figure 1 shows a panel of photomicrographs depicting pathologic lesions that can be detected and quantified, along with a pathologic readout showing the data that are obtained for each patient and used for routine diagnostic practices at the University of Kentucky. The potential benefits of quantitative digital pathologic assessment extend beyond the description of ‘inclusion bodies’ that characterize many neurodegenerative diseases. This is important because many of the comorbid pathologies in the aged human brain involve additional subtypes of disease, including almost universal aging-related vascular, inflammatory, and metabolic changes [37-40]. As such, there are many additional new opportunities to study features of brain histomorphology that have been hitherto constrained by the intrinsic limitations of the human eye for detecting and quantifying geometric characteristics. Morphology of blood vessels - particularly, small blood vessels - provides an important subject area that confounded prior methodologies. Aged brains contain many subtypes of small-vessel changes, including cerebral amyloid angiopathy, arteriolosclerosis, expanded Virchow-Robin spaces, small hemorrhages with hemosiderinladen macrophages, and micro-infarcts [4,41,42]. A novel approach using the ScanScope digital pathologic algorithms was able to make novel discoveries [43], assessing the morphologic characteristics of capillaries (immunolabeled with an antibody raised against CD34) and arterioles (immunolabeled with an antibody raised against alpha-smooth muscle actin). This method was applied to demonstrate, in quantitative fashion, that hippocampal sclerosis of aging in humans [44] is associated with arteriolosclerosis in areas outside of the hippocampus [43]. This analysis would be difficult otherwise, given the lack of a rigorous universal definition of arteriolosclerosis. Figure 2 is a panel to illustrate some of the parameters that can be gleaned by the software when analyzing sections that are immunostained for small-blood vessel profiles. Additionally, the new technology was applied to query neuroinflammation in animal models [45] by analyzing inflammatory cells in mice brains. These experiments included assessment of both quantitative neuroinflammatory changes (number of astrocytes or macrophages in a tissue) and qualitative changes (macrophage activation was addressed by querying macrophages in different morphologic states). Finally, digital pathologic methods enable the study of larger areas of the brain and photomontages to depict multiple pathologic features in those brain areas [46]. Despite the benefits of the digital pathology methods, there are potential drawbacks. The cost of digital pathology could be problematic for some centers and hospitals: hundreds of thousands of dollars for a machine that also requires costly service contracts for future use (currently for the Aperio ScanScope used at the University of Kentucky, the service contract costs over $30,000 USD per year). Furthermore, for a longitudinal study, there is always the question of whether that system will remain well supported by the manufacturer and whether future work will be directly comparable after the inevitable changes in technology. Whole slide analysis was the theoretical goal, but the massive amount of analysis time this required made this an impractical one. There are also specific areas of difficulty. For example, a challenging pathology to quantitate is the neuritic plaque. Owing to the heterogeneous nature of these lesions, single plaques are challenging to count individually. We note that the difficulty in this regard is also reflected by a general lack of consensus on what exactly defines these lesions (silver stain, thioflavine, or tau immunohistochemistry can be used). Also, there are drawbacks to the quantification of immunohistochemical phenomena because those phenomena can be labile to various technical factors, including fixation time and inevitable variabilities in chromagen development.

### Automated microscopes

Fully automated microscopes coupled to a personal computer (PC) and software represent another possibility to perform large-scale quantitative assessment. By conventional image analysis, photomicrographs are individually imported into the image analysis software, and subsequently adequate thresholds are set for measurement. Automated microscopes, on the other hand, may be entirely controlled by software allowing multiple images to be taken automatically; after a specific area of interest on the histological slide is set, multiple images covering this area are taken automatically and then combined into a single large image which is used for measurement (see [47,48]). As images need not be imported individually, this automated method is time-saving. Using this methodology, we could demonstrate that only the amount of neuronal cell loss in the substantia nigra correlated with reduced striatal ^123^I-FP-CIT SPECT (single-photon emission computed tomography) uptake but that the amount of hyperphosphorylated tau, Aβ, and α-synuclein in both striatum and substantia nigra had no influence on striatal ^123^I-FP-CIT SPECT uptake [47]. These findings were possible only by using a quantitative methodology as semi-quantitative assessment shows ‘severe’ nigral cell loss and ‘severe’ amounts of hyperphosphorylated tau, Aβ, and α-synuclein pathology in most cases, making it impossible to detect any differences with regard to the amount of pathology in this study cohort. Recently, we could also demonstrate in human brain tissue that the amount of hyperphosphorylated tau pathology correlates with that of pyroglytamylated Aβ but that no respective correlation was observed between hyperphosphorylated tau and non-pyroglutamylated Aβ [48]. These findings suggest that pyroglytamylated Aβ plays a crucial role in the pathogenesis of AD. Automated microscopes can also be used to quantify pathology on tissue microarrays (TMAs); of note, TMA methodology is often used in cancer research in which one slide contains samples of many different cases. However, in the Newcastle Brain Tissue Resource (Newcastle University, UK), these TMAs are used to assess 40 different regions from any given case. Samples for TMAs from prefrontal (BA9), frontal (BA8), cingulate (BA32/24), motor (BA4), parietal (BA40/22), occipital (BA17), temporal (BA21), and entorhinal (BA28/27) cortices are taken from paraffin-embedded tissue blocks (previously used for conventional neuropathological assessment) by using a 3-mm tissue sampler (Tissue-Tek Quick-Ray TMA System; Sakura, Torrance, CA, USA), and a single, regular-sized (40 × 30 × 5 mm) paraffin block containing all 40 samples is produced. Sections from this TMA block are routinely stained with antibodies against hyperphosphorylated tau, Aβ, and α-synuclein but are available for other immunohistochemical stains as well. To assess TMAs quantitatively, a so-called macro (that is, executive chain of commands) is created by using the image analysis software, NIS Elements (Nikon, Tokyo, Japan); 40 coordinates which correspond to the location of the TMA samples on the slide are set. The fully motorized Nikon 90i microscope is controlled entirely by PC/software, and with a 40× objective (400× magnification), the first acquisition of 3 × 3 images is performed in the center of the first TMA sample (top left). Image analysis is then performed automatically on the combined image, which represents an area of 1.7 mm^2^, using standardized thresholds: red-green-blue thresholds that determine the pixels that are included in the binary layer used for measurement are standardized separately for each immunostain (that is, hyperphosphorylated tau, Aβ, and α-synuclein). We set the thresholds at a level that is reached by immunopositive pathological structures only (that is, NFTs, neuropil threads, Aβ plaques/depositions, and LBs/neurites), but unspecific background staining and structures that do not show immunopositivity (for example, corpora amylacea) do not reach the threshold and thus are not included into the measurement.

The obtained data are automatically stored in a database. The microscope stage then moves automatically to allow image acquisition of the next TMA sample, and the procedure is repeated until images of all 40 samples are measured. Of note, before each image acquisition, autofocus is performed. The assessment of one TMA takes approximately 30 minutes and therefore is suited to be used in a routine setting. Of note, only the database containing the values of the areas covered by immunopositivity are kept on the PC/storage medium, whereas the images that were used for measurement are deleted (the original slides can be re-assessed if necessary). Hence, no extensive storage capacity is needed to keep the data. Using this methodology, we currently assessed over 100 post-mortem brains, including AD, LB disease, and controls. One interesting finding was that the percentage area covered by immunopositivity (hyperphosphorylated tau, Aβ, and α-synuclein) differed considerably within semi-quantitative categories, in particular in areas that were semi-quantitatively scored ‘severe’, where, for example, the percentage area for hyperphosphorylated tau immunopositivity ranged from 10% to over 30%. This further highlights the need for quantitative data in studies aiming to identify subtle and novel clinico-pathological phenotypes which may be characterized by the simultaneous presence of multiple pathologies.

## Conclusions

Some recent discoveries were made possible only by using quantitative methodologies for the assessment of neuropathological lesions. We described some of the new methodologies that allow such a quantification at high throughput, but more methodologies are currently being developed. These techniques will enable the identification of new clinico-pathological phenotypes that reflect cerebral multi-morbidity of the aging brain. It is hoped that future studies identify specific clinical signs or biomarkers that more specifically point toward specific underlying neuropathologies, with respect to both their quantity and quality. Such studies are indeed warranted to allow an accurate stratification of patients in clinical trials and to further elucidate possible interactions between different pathological processes in the aging brain.

## Note

This article is part of a series on *Cerebral multi-morbidity of the aging brain* edited by Johannes Attems and Julie Schneider. Other articles in the series can be found at http://alzres.com/series/cerebral_multimorbidity.

## Abbreviations

ACT: Adult changes in thought
AD: Alzheimer’s disease
ADNC: Alzheimer’s disease neuropathologic change
Aβ: Amyloid-beta
LB: Lewy body
MRI: Magnetic resonance imaging
NFT: Neurofibrillary tangle
PC: Personal computer
SPECT: Single-photon emission computed tomography
TDP-43: Transactivation response DNA binding protein 43 kDa
TMA: Tissue microarray
